# Management of Implantable Cardiovascular Devices in Patients Undergoing Radiotherapy

**DOI:** 10.3390/diagnostics16040578

**Published:** 2026-02-14

**Authors:** Martina Nesti, Maria Laura Canale, Stefano Oliva, Simona Giubilato, Carlo Pignalberi, Rossella Troccoli, Antonio Di Monaco, Irma Bisceglia, Fabio Turazza, Claudio Bilato, Furio Colivicchi, Massimo Grimaldi, Fabrizio Oliva

**Affiliations:** 1 Cardiologia Interventistica, Fondazione Toscana Gabriele Monasterio, 56126 Pisa, Italy; 2 U.O.C. Cardiologia-UTIC, Ospedale Versilia, Azienda USL Toscana Nord-Ovest, 55041 Lido di Camaiore, Italy; marialaura.canale@uslnordovest.toscana.it; 3 U.O.S.D. Cardiologia di Interesse Oncologico, IRCCS Istituto Tumori “Giovanni Paolo II”, 70124 Bari, Italy; stefanoliva66@gmail.com; 4 U.O.C. UTIC-Cardiologia ed Emodinamica, AOE Cannizzaro, 95126 Catania, Italy; simogiub@hotmail.com; 5 U.O.C. Cardiologia Clinica e Riabilitativa, Presidio Ospedaliero San Filippo Neri, ASL Roma 1, 00135 Roma, Italy; c.pignalberi@libero.it (C.P.); furio.colivicchi@aslroma1.it (F.C.); 6 Cardiologia Ospedaliera, Azienda Ospedaliero-Universitaria Consorziale Policlinico di Bari, 70124 Bari, Italy; rossella.troccoli@gmail.com; 7 U.O.C. Cardiologia, Ospedale Generale Regionale “F. Miulli”, Acquaviva delle Fonti, 70021 Bari, Italy; a.dimonaco@gmail.com (A.D.M.); m.grimaldi@miulli.it (M.G.); 8 Servizi Cardiologici Integrati, Dipartimento Cardio-Toraco-Vascolare, Azienda Ospedaliera San Camillo Forlanini, 00152 Roma, Italy; irmabisceglia@gmail.com; 9 Independent Researcher, 20162 Milan, Italy; turazzafabio@gmail.com; 10 U.O.C. Cardiologia, Ospedali dell’Ovest Vicentino, Azienda ULSS 8 Berica, 36100 Vicenza, Italy; claudio.bilato@aulss8.veneto.it; 11 Cardiologia 1-Emodinamica, Dipartimento Cardiotoracovascolare “A. De Gasperis”, ASST Grande Ospedale Metropolitano Niguarda, 20162 Milan, Italy; fabri.oliva@gmail.com; 12 ANMCO—Associazione Nazionale Medici Cardiologi Ospedalieri, 50121 Firenze, Italy; 13 Fondazione per il Tuo Cuore—Heart Care Foundation, 50121 Firenze, Italy

**Keywords:** radiotherapy, pacemaker, ICD, cancer, electrophysiology, risk stratification

## Abstract

The increasing coexistence of oncological disease and cardiovascular implantable technology poses complex clinical challenges that require close collaboration among cardiologists, electrophysiologists, radiation oncologists, and medical physicists. A structured, systematic, and multidisciplinary approach is essential for the safe management of cancer patients with a cardiac implantable electronic device (CIED) undergoing radiation therapy. This document reports a consensus statement issued by the Italian Association of Hospital Cardiologists (ANMCO), aiming to provide practical guidance for clinicians involved across the entire care continuum of this high-risk patient population. A comprehensive pre-treatment evaluation is strongly recommended, including detailed assessment of the type of radiotherapy, treatment planning parameters, device characteristics, and patient-specific cardiac conditions, to minimize the risk of CIED malfunction. Particular emphasis is placed on risk stratification before radiation therapy, as well as on the appropriate timing and modality of cardiological assessments during treatment and in the post-therapy follow-up phase. The document offers an overview of oncological electrophysiology and the mechanisms of radiation-induced damage to cardiac devices with the goal of supporting standardized, safe, and effective clinical practice in this evolving field.

## 1. Introduction

Cancer and cardiovascular disease are both degenerative conditions that become more prevalent with increasing age, as advanced age is a significant shared risk factor for the development of these diseases. As a result, the incidence of both conditions rises markedly in older populations [1].

In recent years, the use of electronic cardiac devices has expanded beyond the original scope of simple pacemaker implantation in elderly patients. Now, more complex devices such as defibrillators and combined biventricular pacing and defibrillation systems—collectively referred to as implantable cardiac electronic devices (CIEDs)—are being used in a broader range of cardiology patients. This is particularly notable in the management of heart failure, where these advanced devices play an essential role.

Simultaneously, radiotherapy (RT) has remained a cornerstone of cancer treatment, being administered to approximately half of all oncology patients. The growing prevalence of both CIEDs and RT has led to an increasing number of patients with CIEDs requiring radiotherapy as part of their cancer management [2].

This convergence presents important clinical challenges, specifically the risk of electrical interference caused by RT that may affect the function of CIEDs. While modern devices are engineered with improved protection against external interference, they could be paradoxically more sensitive to RT than earlier generations of devices.

In most instances, the risk of interference from RT does not result in permanent device damage or pose a serious threat to patient safety.

The use of neutron-producing RT beam energies is the strongest predictor of CIED malfunction and should be avoided where possible. However, as is often the case in the field of cardio-oncology, risk stratification remains paramount. A thorough pre-treatment assessment of cardiovascular risk is essential for planning appropriate clinical and instrumental monitoring, especially for patients undergoing chest RT or when the CIED is located within the radiotherapy treatment field [3].

This document aims to offer practical guidance to cardiologists, radiotherapists, oncologists, and radiotherapists in managing patients with CIEDz who require RT, particularly in settings where an electrophysiologist may not be available for consultation. This document was developed as an expert consensus statement promoted by the Italian Association of Hospital Cardiologists (ANMCO). The writing panel included cardiologists, radiotherapists, oncologists, and radiotherapists with recognized expertise in cardio-oncology, CIEDs and RT. Experts were selected based on their clinical experience, involvement in national scientific societies and working groups, and scientific contributions in the field.

The consensus was reached through iterative multidisciplinary discussions among the authors, integrating clinical expertise with a critical appraisal of the existing literature. A formal consensus methodology, such as a Delphi process or structured voting, was not applied. Instead, agreement was achieved through shared discussion and revision of the manuscript until a common position was reached.

During the preparation of this manuscript/study, the authors used ChatGPT, GPT-5.2 Instant for creating graphical abstract. The authors have reviewed and edited the output and take full responsibility for the content of this publication.

The recommendations are based on a narrative review of the literature, including international guidelines, consensus documents, and key clinical and experimental studies addressing the interaction between RT and CIEDs. During the preparation of this manuscript, the authors used ChatGPT, GPT-5.2 Instant for creating the graphical abstract. The authors have reviewed and edited the output and take full responsibility for the content of this publication.

Given the limited availability of high-level evidence and randomized data in this field, a formal grading of the level of evidence was not performed. As such, the recommendations primarily reflect expert opinion informed by the best available evidence and their overarching goal is to promote closer multidisciplinary collaboration between specialists to optimize patient care in this complex clinical scenario.

## 2. Pre-Treatment Risk Stratification

During the development of treatment plans for malignancies, radiation oncologists should collaborate with cardiologists–electrophysiologists specialized in CIEDs and inform patients about the potential risk of CIED malfunction during RT.

**Figure 1:**
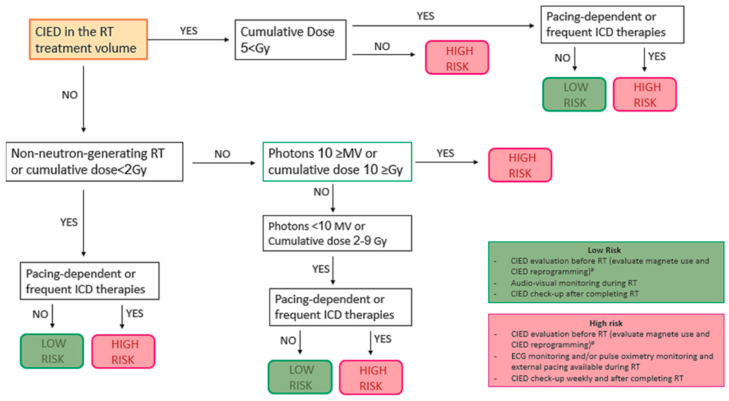
An algorithm for assessing the risk of implantable cardiac electronic device (CIED) malfunction during radiation therapy (RT). ICD, implantable cardiac defibrillator; PM, pacemaker. # Perform a CIED evaluation if the last one was performed more than 3 months ago. Consider magnet placement or CIED reprogramming if the patient is PM-dependent, if the device battery is nearing the end of its life cycle, or if the patient has an ICD (magnet use can be avoided in patients with multiple ICD interventions). In patients with ICD and PM dependence, consider asynchronous programming. Deactivate the sensor in patients with rate-responsive PM.

For all patients undergoing RT, a thorough cardiovascular history and detailed information about the CIED should be obtained, including a review of the latest electronic check or most recent remote transmission. It is essential to determine the patient’s specific risk before starting RT, as patients with high-risk characteristics require close monitoring of device function during treatment. Figure 1 outlines how to assess the risk of CIED malfunction during RT.

A multidisciplinary team should consider each of the following points when evaluating risk [4,5,6,7]:

▪ **Device type**: Implantable cardioverter–defibrillators (ICDs) are more sensitive to ionizing radiation than pacemakers (PMs) due to internal circuit composition and are at higher risk of oversensing and inappropriate shocks [5,8].
▪ **Pacemaker dependency**: Patients without spontaneous ventricular activity or with non-tolerable low heart rates are considered PM-dependent and at high risk of asystole if RT interferes with pacing.
▪ **Estimated absorbed dose**: The medical physicist should estimate the cumulative dose absorbed by the CIED as part of treatment planning. In general, the risk of CIED malfunction increases when the absorbed dose exceeds 5 Gy, which mainly occurs when the planned RT target includes the chest, neck, or proximal upper extremity [3]. Typically, the absorbed dose is <2 Gy if the radiation field is more than 5 cm away from the CIED [6].
▪ **Radiation energy**: Radiation that leads to neutron emission is most likely to damage CIED, even when directed at distant sites below the diaphragm. Photon energy >10 MV, electron energy >20 MeV, and proton therapy are considered high-risk for CIED malfunction due to potential neutron emission. Therefore, treatments that do not involve neutron emission are preferable for patients with a CIED [8].
▪ **Treatment field proximity**: A CIED should not be within the RT field. If unavoidable or high energies are needed, removal or repositioning of the device may be considered. This option is rarely necessary and the risks of infection and surgical complications must be assessed.
▪ **Patient-specific factors**: Immunosuppression, chemotherapy, and comorbidities influence the risk–benefit ratio of device relocation.

Once the risk of CIED malfunction has been assessed, patients should be managed according to their risk level:

**Low-risk patients** (e.g., non-dependent pacemaker users, device far from the radiation field, low cumulative dose, no neutron emission) may undergo RT with standard precautions and periodic monitoring.

**High-risk patients** (e.g., PM-dependent, ICD users, high-dose exposure, neutron-emitting treatments) should be managed with a multidisciplinary approach, including continuous monitoring during RT, emergency protocols, and possibly relocating the device if feasible.

## 3. Preventive Strategies

Based on risk stratification, tailored preventive measures are needed to ensure patient safety during RT.

### 3.1. Device Management During RT

- **PM**: For patients with rate-responsive PMs, temporary deactivation of the sensor during RT should be considered.
- **ICD**: For patients with ICDs, deactivation of antitachycardia therapies is recommended, either through device reprogramming or by applying a magnet over the device [8,9,10,11]. However, antitachycardia therapy deactivation can be avoided in patients with multiple ICD interventions.

### 3.2. Magnet Use

There are limited data on the necessity and benefits of magnets or reprogramming PMs or ICDs during thoracic RT. Magnet use or reprogramming may be considered for patients who are PM-dependent, have devices nearing battery depletion, or have ICDs [7,8,10,11,12].

Magnets have different effects on PMs and ICDs. In PM patients, placing a magnet over the CIED pocket disables the sensing of cardiac activity, ensuring fixed-rate asynchronous pacing regardless of any cardiac or non-cardiac electrical signals. In PM patients without spontaneous ventricular activity, this prevents oversensing due to electromagnetic interference, which could otherwise lead to asystole. In ICD patients, a magnet disables antitachycardia therapies, preventing inappropriate shocks from oversensing. However, PM functions are usually unaffected, and in ICD patients who are PM-dependent, asynchronous pacing mode should be programmed to ensure consistent ventricular stimulation.

As previously discussed, although the risk of ICD oversensing is extremely low, magnet use is recommended to disable antitachycardia therapies [11,13]. In some patients with frequent ICD interventions, magnet use may be avoided. In summary, personalized planning of RT strategies is essential, and the cardiologist must clearly define magnet use for each specific case.

### 3.3. Monitoring Protocols

During RT, staff should be able to visually observe and communicate with all patients throughout the treatment session, although this recommendation is not present in all guidelines.

High-risk patients should have continuous heart rate monitoring using the pulse oximetry and immediate access to a 12-lead electrocardiogram, if necessary, external pacing and defibrillation devices, and personnel trained in advanced cardiac life support/cardiopulmonary resuscitation in an emergency. Continuous electrocardiographic monitoring during RT is generally not necessary, but may be considered in patients with ICDs who have undergone previous treatment for ventricular arrhythmias [7,8,11]. The presence, during RT sessions, of an electrophysiologist (or a technician/nurse experienced in CIED management) for urgent CIED interrogation and programming should be considered necessary only in very selected cases of high-risk patients, and an “on-call” electrophysiologist (available on call within minutes) may be considered.

### 3.4. Relocating CIED

As previously stated, CIEDs should not be placed directly within the RT treatment area. If the device lies within the planned radiation beam path, it may interfere with effective tumor treatment. Photon beam energy should be kept below 10 MV, as the risk of CIED malfunction or damage increases beyond this threshold [7,9,10]. If higher doses are required or the CIED cannot be excluded from the beam, removal and relocation of the device away from the beam should be considered, although this is rarely necessary [6,7,9,10].

The primary reason for relocating the CIED is to allow for effective RT of the tumor, but the potential for RT-induced malfunction or damage requiring device replacement should also be considered. However, explantation and reimplantation carry significant risks, including infection, which is particularly relevant in patients undergoing chemotherapy or those who are immunocompromised [6,7,9,10]. For most patients undergoing definitive tumor treatment, the risk–benefit ratio usually favors relocation. In contrast, for patients receiving palliative RT or with significant comorbidities, relocation may be avoided. Moreover, since CIED malfunction mainly occurs due to neutron contamination, relocating the device to the contralateral side is not necessarily protective, as neutrons can penetrate significant distances. These decisions must be made by a multidisciplinary team in collaboration with the patient. Relocation is not recommended for devices receiving a cumulative incidental dose below 5 Gy, where the risk is considered negligible.

## 4. Management and Follow-Up

The incidence of malfunction of CIEDs following RT is extremely low and, in most cases, not clinically relevant. Nevertheless, exposure to ionizing radiation can occasionally lead to changes in device parameters such as variations in sensing, pacing threshold, and lead impedance, premature battery depletion, and, more rarely, spontaneous resets or reboots of the device, which may increase susceptibility to electromagnetic interference [10,13].

Moreover, RT can induce local cutaneous and subcutaneous fibrosis, leading to tissue alterations that may necessitate pocket revision procedures [14].

### 4.1. Evidence from the Literature

In a systematic review of the literature encompassing studies published between 2002 and 2021 on CIED patients undergoing RT, an overall rate of malfunction of 6.6% (135 out of 3121 patients) was reported, though only three cases were considered potentially life-threatening (two inappropriate shocks and one ventricular tachycardia). These events occurred mainly during treatment and were rarely observed in the long-term follow-up. The type of treatment (hadron therapy), the device type (ICD or CRT-D compared with pacemaker or CRT-P), and a maximum delivered dose to the CIED greater than 2 Gy were the main predictors of malfunction in this population [15].

A retrospective analysis published in 2015 confirmed a similar incidence (7%) in the absence of life-threatening events and further indicated that non-neutron-producing RT appeared to be safer for patients carrying cardiovascular devices [16].

In a more recent study by Karimzad et al., device malfunctions were even rarer (1.1%), likely reflecting technological improvements in CIED design. These events were associated with neutron-producing radiation (proton therapy or photon energy >10 MV). Device type and radiation field location did not appear to influence the likelihood of device resets. Notably, transient signal interference or oversensing—issues previously encountered in older devices—were not observed with contemporary systems [3].

### 4.2. Guideline Overview and Practical Implications for Clinical Practice

To prevent the occurrence of CIED malfunctions, albeit rare, several national and international consensus documents have been published addressing optimal management and follow-up strategies. These include the AAPM TG-203 guidelines [17], the HRS consensus [8], the Italian AIAC/AIRO/AIFM joint consensus document [11], the EHRA consensus on electromagnetic interference [12], and the 2022 ESC Guidelines [7]. All these documents converge on the recommendation to plan clinical and instrumental follow-up based on an individual risk assessment.

The recommendations, however, have evolved over time. The German guidelines published in 2015 [18] suggested performing a device check after each RT fraction, regardless of the estimated risk. In contrast, the ESC 2022 guidelines [7] advocate a risk-based schedule, modulating the frequency of follow-up according to pacemaker dependency and the cumulative radiation dose delivered to the device. The Italian consensus document [11] proposes an intermediate position, recommending device interrogation before, during, and after the cycle of RT in low-risk patients, alongside continuous visual and vocal monitoring during irradiation. This evolution reflects growing evidence that has progressively supported a reduction in the frequency of device checks without compromising patient safety.

The number and timing of device checks should consider several factors, including the type of RT (high-energy neutron-producing versus conventional photon therapy), beam energy, device location relative to the radiation field, and the patient’s dependency on pacing support. It is generally recommended to avoid direct irradiation of the device whenever possible and to maintain the cumulative dose below 2 Gy for pacemakers and 1 Gy for ICDs [17]. In addition, continuous visual and vocal monitoring of the patient is strongly advised during the RT session.

According to the ESC Guidelines [7], post-RT follow-up depends on a physician-led risk stratification process that classifies patients as low or high risk based on radiation dose and pacemaker dependency. The electrophysiologist plays a key role in both categories, albeit with differing follow-up frequencies.

Low-risk patients—those who are not pacemaker-dependent and whose device receives a low radiation dose—should undergo device interrogation only before and after RT. High-risk patients—either pacemaker-dependent or exposed to a cumulative device dose exceeding 5 Gy (if the device lies outside the treatment field) or 10 Gy (if within the field)—should undergo weekly ECG monitoring and device interrogation until the end of the RT program. The HRS consensus [8] adopts a similar approach, recommending weekly device checks in cases involving neutron-producing RT or pacemaker-dependent patients, while in all other scenarios a single post-treatment evaluation is considered sufficient (Table 1; Supplementary Materials, Table S1).

Remote monitoring can also be considered when available and clinically appropriate. Finally, it is advisable to adhere to manufacturer-specific recommendations, as follow-up protocols may vary according to device type and exposure conditions [19] (Table 2).

### 4.3. Management of New-Generation CIEDs

In recent years, innovative technologies have been developed for the treatment of conduction disorders. Subcutaneous implantable cardioverter–defibrillators (S-ICDs) and leadless pacemakers are increasingly used in selected patient populations to reduce complications, particularly the risk of device-related infections. In these systems, the absence of transvenous leads reduces the risk of radiation-induced lead damage; however, the pulse generator of S-ICDs is often positioned close to thoracic irradiation fields, potentially increasing direct radiation exposure. The potential complications related to radiotherapy in S-ICDs are similar to those observed with conventional devices, including device resets and sensing disturbances. Conversely, leadless pacemakers, which are implanted entirely within the right ventricle, are theoretically less likely to receive a significant radiation dose, although available clinical data remain limited and only clinical cases are described in the literature [20,21]. Owing to this lack of evidence, the recommended management of new-generation CIEDs does not differ from that of traditional transvenous devices [11]. Pre-treatment planning should aim to minimize radiation dose to the device whenever feasible, and device interrogation before and after radiotherapy is recommended, with additional monitoring in pacing-dependent patients. Given the limited clinical evidence, individualized risk assessment and multidisciplinary management remain essential.

## 5. Multidisciplinary Approach

Radiotherapy in patients with CIEDs [22], including pacemakers and implantable cardioverter–defibrillators, requires an organizational model based on the synergistic involvement of multiple professionals in order to ensure both treatment efficacy and patient safety [11]. These cases pose significant challenges due to the potential interaction between therapeutic ionizing radiation and implanted cardiac device. Such interactions necessitate careful risk assessment prior to treatment and coordinated management throughout the entire therapeutic pathway.

The increasing survival of patients with cancer, who often have coexisting cardiovascular comorbidities, combined with the growing prevalence of CIEDs in the general population, makes this clinical setting increasingly common. The coexistence of oncologic and arrhythmic diseases requires an approach that prevents detrimental interference between radiotherapy and cardiac devices, preserving the therapeutic efficacy of radiotherapy and the uninterrupted function of cardiac pacing or defibrillation, which is often life-sustaining.

**Figure 2:**
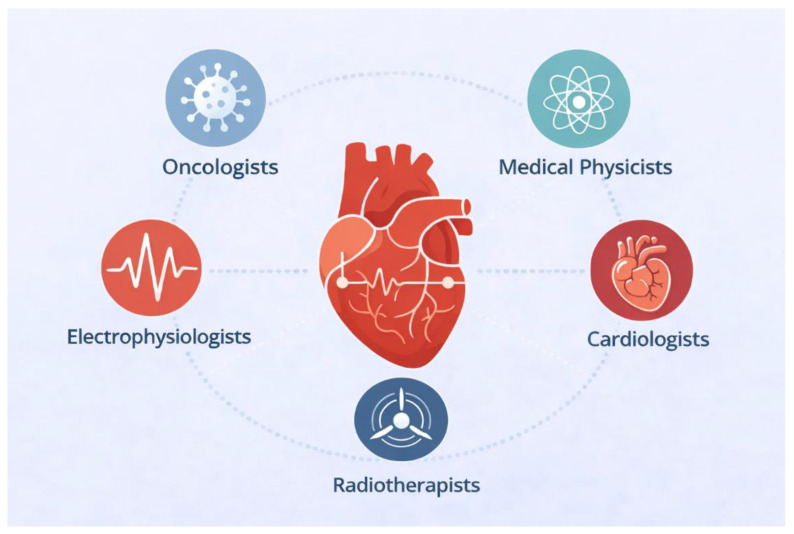
The multidisciplinary team in the management of patients with cardiac implantable electronic devices during radiotherapy.

A structured multidisciplinary approacj is therefore fundamental (Figure 2).

Continuous and formal communication among all professionals involved allows for accurate risk stratification, appropriate preventive strategies, and timely intervention in the case of complications during the treatment process [22]. This collaborative model is increasingly recognized as the standard of care in modern oncology for achieving truly personalized management and a safe therapeutic approach.

Within the team, oncologists and radiation oncologists play a pivotal role in establishing the indication for radiotherapy and in defining the treatment plan. Their responsibility is to select the most appropriate regimen by considering the type of malignancy, dose distribution, and the need to preserve device functionality, while reducing the risk of electromagnetic interference and direct damage to CIED components. The radiation oncologist, in particular, should classify the device as an organ at risk (OAR) and adopt technical solutions to limit both direct and incidental exposure of the device, involving cardiology specialists early in the planning process [6].

The medical physicist provides essential support by estimating the radiation dose absorbed by the device, accounting for both direct exposure and secondary radiation from imaging and treatment scatter. These measurements are fundamental for individual risk assessment and for guiding the selection of appropriate protective measures.

**Figure 3:**
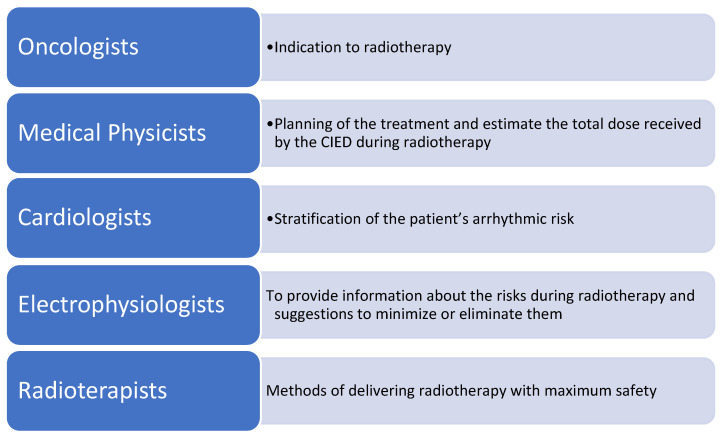
The roles and the responsibilities of each member of the multidisciplinary team.

The cardiologist with expertise in CIED management, or the electrophysiologist, assumes a crucial role in this context. These specialists must first reconstruct the arrhythmic profile of the patient by reviewing the indication for device implantation, the type of CIED, lead configuration, recent ICD therapies, and pacemaker dependency. Particular attention must be paid to the timing of device checks, including whether remote monitoring is active, to ensure adequate surveillance before, during, and after radiotherapy [4]. The cardiology assessment plays a decisive role in clinical risk stratification, integrating radiotherapy-specific information and determining the appropriate level of monitoring for each patient. The cardiologist or electrophysiologist also provides guidance on potential device reprogramming, recommends protective intra-procedural strategies, and instructs the team on how to respond to signs of malfunction. This includes defining a clear emergency protocol for the prompt management of arrhythmic events or device failure. The roles and the responsibilities of each member of the multidisciplinary team are summarized in Figure 3.

Clear communication with the patient is another essential aspect of care. Patients should be informed of the potential risks, trained to recognize warning symptoms such as dizziness, presyncope, or syncope, and encouraged to report them without delay. Radiotherapy nurses and technicians play an active role in continuous clinical monitoring and early detection of complications.

Ultimately, the multidisciplinary approach ensures that diverse professional expertise converges toward a shared goal, achieving optimal oncologic outcomes while preserving the arrhythmic protection provided by the CIED. Ongoing innovations in cardiac device design and radiation delivery techniques are expected to further enhance this interspecialty collaboration [23].

Looking ahead, artificial intelligence (AI)-based decision support systems may refine risk assessment and guide personalized management in complex cases. Moreover, telemedicine and virtual multidisciplinary boards could extend specialized electrophysiology expertise to smaller centers, ensuring equitable, high-quality care across diverse healthcare settings.

## 6. Future Perspectives

At present, it remains challenging to predict the future of clinical management for patients with a CIED undergoing RT. Increasing technological integration and more efficient multidisciplinary collaboration are likely to represent highly promising directions for future development. In this complex clinical setting, the evolution of multidisciplinary teams is expected to include a progressively greater involvement of AI. AI may prove useful in supporting experts in the interpretation of complex cases and in the selection of the most effective and safest therapeutic options.

Another crucial aspect will likely be the expansion of telemedicine, which will enable smaller centers to consult specialists from referral institutions regarding complex cases, thereby ensuring optimal patient care.

An open issue may persist concerning the higher susceptibility of newer-generation devices to external interference compared with older models. This increased vulnerability is mainly attributable to the use of complementary metal-oxide semiconductor (CMOS) technology, characterized by thinner layers and higher component density. Unfortunately, current knowledge regarding the relationship between the amount of CMOS circuitry and the radiation sensitivity of a CIED remains incomplete, and clear radiation dose thresholds for each device category have yet to be established.

Future advances in treatment techniques may also change the overall landscape, potentially reducing CIED interference thanks to the lower tissue toxicity associated with newer radiotherapy modalities such as intensity-modulated radiotherapy (IMRT) and volumetric modulated arc therapy (VMAT). However, proton therapy—while offering the unique advantage of concentrating energy within the target area—produces scattered thermal neutrons that may induce a high risk of “soft errors” in the device, regardless of its position. Moreover, comprehensive data are still lacking on the effects of RT on the latest devices, such as leadless pacemakers and S-ICDs. Notably, an in vitro study suggested that S-ICDs might carry a higher risk of radiation-induced damage compared with transvenous systems.

In summary, although international guidelines continue to evolve, future perspectives should focus on improving personalized risk management through advanced technologies (AI and telemedicine) and addressing current knowledge gaps—particularly by defining precise radiation damage thresholds and investigating the impact of RT on next-generation devices and emerging treatment modalities.

## Figures and Tables

**Table 1:** Frequency and type of follow-up in the various guidelines/consensus statements/position papers (CIED: implantable cardiac electronic device; ICD: implantable cardiac defibrillator; RT: radiotherapy).

	German Guidelines [18]	ESC Guidelines 2022 [7]	Italian Consensus [11]	EHRA Consensus [12]	HRS Consensus [8]
***Low Risk***	- Weekly CIED check - Audiovisual contact - ICD: magnet application or pacing deactivation	- CIED check before and after RT	- CIED interrogation before, during, and after the cycle of RT with continuous visual and vocal monitoring during irradiation	- Baseline check and post-RT assessment	- Single post-RT evaluation
***Intermediate Risk***	- Weekly CIED check - Availability of an emergency cart with defibrillator and external pacemaker - Availability of the emergency team within 10 min	Not applicable	- CIED assessment before and after RT, with weekly checks during treatment	- Baseline, interim, and post-RT CIED checks (or remote monitoring)	Not applicable
***High Risk***	- Reassess the need for RT - CIED check within 24 h after the end of each treatment - Continuous ECG monitoring during RT	- Pre-RT CIED check - Availability of ECG and SpO2 monitoring - Weekly CIED check	- CIED assessment before and after RT, with weekly checks during treatment - Continuous monitoring during RT therapy, with qualified personnel and equipment available for the management of arrhythmic emergencies	- Baseline and weekly CIED checks during RT - Post-RT CIED check	- Weekly device checks

**Table 2:** Recommendations for the management of follow-up in patients with a cardiac implantable electronic device undergoing radiation therapy, according to the device manufacturer.

	Medtronic	St. Jude	Boston	Biotronik
Device follow-up after radiotherapy	Yes	Yes	Yes, including close monitoring of device function	Yes, including an additional short-term follow-up after radiotherapy

## Data Availability

No new data were created or analyzed in this study. Data sharing is not applicable to this article.

## Supplementary Materials

The following supporting information can be downloaded at: https://www.mdpi.com/article/10.3390/diagnostics16040578/s1, Table S1. Radiation dose thresholds for cardiac implantable electronic devices according to device type, risk category, and guideline source.

## Abbreviations

The following abbreviations are used in this manuscript:

CIED	Cardiac Implantable Electronic Device
RT	Radiotherapy
ICD	Implantable Cardioverter–Defibrillator
PM	Pacemaker
AI	Artificial Intelligence
CMOS	Metal-Oxide Semiconductor
VMAT	Volumetric Modulated Arc Therapy
S-ICD	Subcutaneous Implantable Cardioverter–Defibrillator
