# Supplementary material for: Management of Implantable Cardiovascular Devices in Patients Undergoing Radiotherapy
Source: Diagnostics (Basel). 2026 Feb 14;16(4):578. doi: 10.3390/diagnostics16040578 (PMC12939585; doi:10.3390/diagnostics16040578)
Supplement: Supplementary file 1 [file diagnostics-16-00578-s001.zip › diagnostics-4111975-supplementary.pdf]

Table S1. Radiation dose thresholds for cardiac implantable electronic devices according to device type, risk category, and guideline source

Device Type	Device Position	Dose Threshold	Clinical Meaning	Guideline / Source
**Pacemaker (PM)**	Outside RT field	<2 Gy	Generally considered safe; standard precautions	AAPM TG-203; ESC 2022
**Pacemaker (PM)**	Outside RT field	2–5 Gy	Increased risk; closer monitoring recommended	ESC 2022; Italian Consensus
**PM**	Outside RT field	>5 Gy	High-risk; intensified monitoring required	ESC 2022
**PM**	Within RT field	Up to 10 Gy	Acceptable only if unavoidable; high-risk scenario	ESC 2022
**ICD**	Outside RT field	<1–2 Gy	Conservative safety threshold	AAPM TG-203; HRS
**ICD**	Outside RT field	2–5 Gy	Moderate risk; risk-based follow-up	ESC 2022; Italian Consensus
**ICD**	Outside RT field	>5 Gy	High-risk; weekly device checks advised	ESC 2022; HRS
**ICD**	Within RT field	Up to 10 Gy	High-risk; consider relocation if feasible	ESC 2022
**Any CIED**	Any position	Any dose with neutron-producing RT	High-risk irrespective of dose	HRS; AAPM TG-203
